# Comprehensive Analysis of G-Quadruplexes in African Swine Fever Virus Genome Reveals Potential Antiviral Targets by G-Quadruplex Stabilizers

**DOI:** 10.3389/fmicb.2021.798431

**Published:** 2021-12-16

**Authors:** Elishiba Muturi, Fei Meng, Huan Liu, Mengwei Jiang, Hongping Wei, Hang Yang

**Affiliations:** ^1^CAS Key Laboratory of Special Pathogens and Biosafety, Center for Biosafety Mega-Science, Wuhan Institute of Virology, Chinese Academy of Sciences, Wuhan, China; ^2^University of Chinese Academy of Sciences, Beijing, China

**Keywords:** ASFV, G-quadruplex (G4), putative G-quadruplex-forming sequence (PQS), G4 ligand, antiviral target

## Abstract

African Swine Fever Virus (ASFV), a lethal hemorrhagic fever of the swine, poses a major threat to the world’s swine population and has so far resulted in devastating socio-economic consequences. The situation is further compounded by the lack of an approved vaccine or antiviral drug. Herein, we investigated a novel anti-ASFV approach by targeting G-Quadruplexes (G4s) in the viral genome. Bioinformatics analysis of putative G-quadruplex-forming sequences (PQSs) in the genome of ASFV BA71V strain revealed 317 PQSs on the forward strand and 322 PQSs on the reverse strand of the viral genome, translating to a density of 3.82 PQSs/kb covering 9.52% of the entire genome, which means that 85% of genes in the ASFV genome have at least 1 PQS on either strand. Biochemical characterization showed that 8 out of 13 conserved PQSs could form stable G4s in the presence of K^+^, and 4 of them could be stabilized by G4 ligands, N-Methyl Mesoporphyrin (NMM), and pyridostatin (PDS) *in vitro*. An enhanced green fluorescent protein (EGFP)-based reporter system revealed that the expression of two G4-containing genes, i.e., P1192R and D117L, could be significantly suppressed by NMM and PDS in 293T cells. In addition, a virus infection model showed that NMM could inhibit the replication of ASFV in Porcine Alveolar Macrophages (PAM) cells with an EC_50_ value of 1.16 μM. Altogether, the present study showed that functional PQSs existent in the promoters, CDS, 3′ and 5′ UTRs of the ASFV genome could be stabilized by G4 ligands, such as NMM and PDS, and could serve as potential targets for antivirals.

## Introduction

African Swine Fever Virus (ASFV) is an arbovirus that causes African Swine Fever (ASF), a highly contagious and fatal hemorrhagic fever affecting domestic pig and wild boars (both species of *Sus scrofa*) (Arias and Sánchez-Vizcaíno, 2002). The ASFV genome consists of 170–193 kb linear double-stranded DNA encoding more than 150 Open Reading Frames (ORFs). ASFV infection induces varying clinical forms of the disease ranging from acute to chronic infections, depending on the host’s characteristics and the virus strain (Tulman et al., 2009). Acute forms of the disease are characterized by high fever, lethargy, respiratory distress, anorexia, nasal discharge, increased pulse rate, skin cyanosis, spontaneous abortion in pregnant sows, vomiting, and diarrhea, with death occurring 7–10 days after the onset of clinical symptoms. The mortality rate is often 100% (Costard et al., 2013). ASFV has severely impacted the global swine industry, as reflected by a hike in pork prices, shut down of export markets, and shortage of swine-derived heparin (Sánchez-Cordón et al., 2018; Vilanova et al., 2019). This situation is further aggravated by the lack of an approved vaccine or antiviral drug for the prevention or treatment of the disease. Countermeasures to contain ASF outbreaks are limited to strict animal quarantine and implementation of stamping-out policies (Gaudreault et al., 2020).

Several studies have reported various types of active anti-ASFV compounds categorized as either direct-acting antivirals or host-targeting antivirals based on their inhibitory mechanism, as well as those whose mechanism of action is yet to be established (Arabyan et al., 2019). These drugs include: (i) nucleoside analogs that mimic endogenous nucleosides and block viral replication by impairing nucleic acid synthesis or inhibiting enzymes involved in the nucleoside metabolism, such as iododeoxyuridine (Gil-Fernández et al., 1979), rigid amphipathic fusion inhibitors (RAFIs) (Hakobyan et al., 2018), and (S)-HPMPA (Arzuza et al., 1988); (ii) Interferons such as porcine IFN-gamma or as combined treatment with IFN-alpha (Esparza et al., 1988; Paez et al., 1990); (iii) Plant-derived compounds including genistein that impairs type II topoisomerase activity and apigenin derivatives such as genkwanin, a tubulin polymerization inhibitor (Freitas et al., 2016; Hakobyan et al., 2019); (iv) antibiotics such as rifampicin and coumermycin A1 that target DNA-directed RNA polymerase and type II topoisomerase, respectively (Dardiri et al., 1971; Salas et al., 1983); and (v) Small interfering RNA and CRISPR/Cas9. SiRNA targeting essential genes A151R and B646 L inhibits viral DNA replication (Keita et al., 2010). CRISPR/Cas9 has been demonstrated to inhibit ASFV by targeting codons 71–78 of the phosphoprotein p30 encoded by the CP204L gene (Keita et al., 2010). ASFV inhibitors with unidentified targets include lambda and kappa carrageenan, resveratrol and oxyresveratrol, lauryl gallate, and some marine microalgae extracts (García-Villalón and Gil-Fernández, 1991; Fabregas et al., 1999; Hurtado et al., 2008; Galindo et al., 2011). However, none of the above compounds has been approved as a treatment for ASFV. There is a dire need to identify or design more inhibitors of ASFV that would provide potential candidates for antiviral evaluation.

While further development of anti-ASFV drugs largely depends on the fundamental knowledge of viral gene properties and functions, comprehensive genomic characterization of ASFV has been obstructed by the complex nature of the virus genome and its lifecycle (Salas and Andrés, 2013; Gaudreault et al., 2020). We explored the value of targeting G-quadruplexes (G4s) in the ASFV genome as an alternative antiviral strategy to circumvent this limitation. G4s are non-canonical nucleic-acid structures formed by guanine-rich sequences that are stabilized through Hoogsteen-type hydrogen bonds base pairing in the presence of monovalent cations such as K^+^ (Burge et al., 2006). G4s are thermodynamically stable and have much slower unfolding kinetics than other DNA hairpin structures (Lane et al., 2008). Mounting evidence suggests their involvement in key biological processes, including transcription, replication, and regulation of gene expression, hence are of great therapeutic interest from a drug development perspective (Rhodes and Lipps, 2015; Li et al., 2021). Targeting G4s has the edge over other DNA structures. For example, it is possible to target G4s in the promoter regions of a gene regardless of the druggability of the gene product. Additionally, there is a likelihood of multiple existing targets since all clinically relevant genes harboring functional G4s are potential druggable targets.

In this study, we comprehensively analyzed the genome-wide presence and distribution of G4s in the ASFV genome. Through bioinformatic prediction and experimental screening, we identified two functionally significant putative G-Quadruplex-forming sequences (PQSs), i.e., PQS-235 and PQS-255, located in D117L and P1192R genes, respectively. Targeting both PQSs with G4-binding ligands N-Methyl Mesoporphyrin (NMM) and pyridostatin (PDS) markedly repressed gene expression levels in 293T cells. Moreover, NMM exhibited a dose-dependent inhibitory effect on the replication of ASFV in Porcine Alveolar Macrophages (PAM) cells. Altogether, the current data showed that targeting G4s in the ASFV genome with G4-stabilizing ligands may present a promising antiviral approach.

## Materials and Methods

### Computational Prediction of Putative G-Quadruplex-Forming Sequences in the African Swine Fever Virus Genome

The complete genome sequences of 42 ASFV isolates were downloaded in FASTA format from the NCBI genome database, and the ASFV BA71V isolate was used as our reference genome. The list of all isolates used in this study is provided in Supplementary Table 1. Coordinates of genomic features, including the coding sequences (CDS), 5′ untranslated regions (5′ UTR), and 3′ untranslated regions (3′ UTR), were inferred as annotated by Cackett et al. (2020). The promoter was estimated as the region spanning 1000 nucleotides upstream of each gene’s annotated transcription start site (TSS).

The reference genome was scanned for PQSs on both the forward and reverse strands using a web-based algorithm software, QGRS Mapper^1^. The PQS search pattern was defined as G _≥__2_N_1–12_G _≥__2_N_1–12_G _≥__2_N_1–12_G _≥__2_, where the G refers to the number of guanine tracts while N refers to either the A, C, T, or G nucleotide. The output provided PQSs in the ASFV genome, including their location, strand sequence, and G-Score that is a predictive score of the folding propensity and stability of the G4 motifs. The number of PQSs in the CDS, 3′ UTR, 5′ UTR, and promoter regions were counted. The density was calculated by dividing the total number of PQSs in the double-stranded ASFV genome by the genome size.

Multiple alignment of the ASFV complete genomes was performed for conservation analysis using MAFFT software^2^. The conservation of PQSs found in the reference genome was calculated using the formula;


C⁢o⁢n⁢s⁢e⁢r⁢v⁢a⁢t⁢i⁢o⁢n⁢r⁢a⁢t⁢e=c⁢o⁢u⁢n⁢t⁢o⁢f⁢t⁢h⁢e⁢g⁢e⁢n⁢o⁢m⁢e⁢s⁢w⁢i⁢t⁢h⁢t⁢h⁢e⁢P⁢Q⁢S⁢i⁢n⁢ 100%⁢i⁢d⁢e⁢n⁢t⁢i⁢t⁢yc⁢o⁢u⁢n⁢t⁢o⁢f⁢a⁢l⁢l⁢t⁢h⁢e⁢c⁢o⁢m⁢p⁢l⁢e⁢t⁢e⁢g⁢e⁢n⁢o⁢m⁢e⁢s×100%

### Preparation of Oligonucleotides

From the predicted PQSs, we selected 13 sequences for biophysical and biochemical characterization (Supplementary Table 2). Our selection was based on the following criteria: (i) PQSs found in the CDS regions of a gene of known function, (ii) a G-score greater than 30, and (iii) a conservation score of ≥98%. All oligonucleotides were synthesized from Sangon Biotech, China, and were received in a lyophilized form. Nuclease-free water was added to the oligonucleotides to prepare a 1 mM stock per strand stock solution. The stock solution was incubated at 4°C overnight to ensure complete solubilization.

### Circular Dichroism

For circular dichroism (CD) analysis, the stock of oligonucleotides was diluted to a final concentration of 5 μM in 10 mM Tris–HCl, pH 7.0, supplemented with 100 mM KCl where necessary. Sealed screw-cap plastic microcentrifuge tubes were filled with enough of the oligonucleotide solution to fill the quartz cuvette. The tubes were placed in a water bath heated to 95°C for 5 min and then cooled gradually to room temperature. CD spectra were recorded on a Chirascan Spectropolarimeter fitted with a Peltier temperature controller using a quartz cuvette of 1 mm optical path length. The operating parameters were set at; 220–320 nm wavelength range, 50 nm/min scanning speed, and 1.0 nm step size with 2 scan repeats. Buffer baseline was recorded using the same parameters and subtracted from the sample spectra before plotting.

For CD melting spectroscopy and ligand binding assays, a similar procedure was followed by the addition of G4-binding ligands NMM and PDS to final concentrations of 5 or 10 μM. Melting transitions were recorded at a wavelength of 265 nm over a temperature range of 25–95°C with a ramping speed of 5°C/min. Melting temperatures (T_m_) values were analyzed using Global 3 Thermal Analysis Software (v3.1.0.3). The data was plotted on graphs expressing molar ellipticity as a function of temperature using GraphPad prism (v9.0.0).

### Construction of Putative G-Quadruplex-Forming Sequences-Enhanced Green Fluorescent Protein-N1 Plasmids

Full lengths of two PQS-harboring genes were inserted upstream of the enhanced green fluorescent protein (EGFP) coding sequence of a pEGFP-N1 vector (Clontech) to investigate the stabilization of G4 structures *in vitro*. Primers used in this study are listed in Supplementary Table 3.

### Effect of G4-Stabilizing Ligands on Putative G-Quadruplex-Forming Sequence *in vitro*

293T cells were cultured in 24-well plates at a density of 1 × 10^5^ cell/well and incubated in DMEM media supplemented with 2% FBS under 5% CO_2_ at 37°C for 24 h. According to the manufacturer’s instructions, the constructs were transfected into the cells using Lipofectamine 3000 reagent (Life Technologies, United States). Cells were then treated with 20 μM each of NMM or PDS. 24 h post-transfection, confocal microscopy was used to evaluate the EGFP expression levels. 4′,6-diamidino-2-phenylindole (DAPI) was used for nuclear staining. Cells transfected with empty pEGFP-N1 vector were used as controls. The fluorescence intensity of each treatment was calculated by corrected total cell fluorescence method and analyzed by one-way ANOVA.

### MTT Assay

The cytotoxicity of NMM in PAM cells was assayed using a tetrazolium-based MTT (3-[4,5-dimethylthiazol-2-yl]-2,5 diphenyl tetrazolium bromide) assay. Briefly, cells were seeded at a density of 1 × 10^5^ cells per well in a 96-well plate. The cells were incubated in RPMI 1640 media supplemented with 10% FBS in a humidified atmosphere containing 5% CO_2_ at 37°C for 24 h. The cells were exposed to a series of concentrations of each compound (0, 1.5625, 3.125, 6.25, 12.5, 25, 50, and 100 μM) and incubated for another 24 h. 10 μl of MTT (5 mg/ml) was carefully added to each well. After 4 h of co-incubation, the supernatant was discarded. 200 μl of DMSO was added and shaken slowly for ∼10 min to resolve the MTT sediment. Optical density was measured at 570 nm using a Synergy H1 microplate reader (BioTek, United States).

### Antiviral Activity of G4-Stabilizing Ligands Against African Swine Fever Virus

A cell-based assay was performed to assess the effect of NMM on the ASFV virus replication. All experiments were carried out in Xiaohongshan Biosafety Level 3 Laboratory of Wuhan Institute of Virology. ASFV Wuhan 2019-2 strain (GenBank: MN393477) was propagated in PAM cells and titrated by standard plaque assay. Briefly, PAM cells were seeded at a density of 2 × 10^5^ cells/well in a 12 well plate a day before infection. 10-fold dilutions of the virus stock were made, ranging from 10^–1^ to 10^–7^. 300 μL of diluted viral stock was added per well in triplicate wells per dilution. The inoculum was left on the cells for 1 h at 37°C and was removed after that. The cells were covered in 1 ml of 1× overlay medium. The Overlay medium was removed 5 days later, and the cell monolayer was washed two times with PBS. The monolayer was fixed with 1 ml 4% paraformaldehyde for 15 min at room temperature. Cells were stained with 0.5 ml of 2% crystal violet solution for 5 min. The stain was removed and washed with PBS. Plates were then washed by immersing in a container filled with tap water. Plaques were counted to determine the titer in PFU/ml by the formula: Average number of plaques/(volume of inoculum plated × dilution factor).

For the antiviral activity assay, PAM cells were seeded at a density of 3 × 10^5^ cells/well in 24 well plates and incubated for 24 h. Cells were then inoculated with ASFV Wuhan 2019-2 strain at a multiplicity of infection (MOI) of 0.1 in the presence of varying concentrations of NMM (0, 0.03, 0.1, 0.3, 1, 3, and 10 μM). At 72 h post-infection, viral nucleic acid was extracted from the cell supernatant using a DNA extraction kit (Qiagen) following the manufacturer’s instructions. The viral yield was quantified by qRT-PCR with primers targeting the P72 gene as described previously (Liu et al., 2021). The primer sequences are P72-F: 5′-TCCTGAAAGCTTATCTCTGCG-3′ and P72-R: 5′-AGATTGGCACAAGTTCGGAC-3′, combined with the probe 5′-FAM-TGAGTGGGCTGCATAATGGCGTT-BHQ1-3′. A non-linear regression analysis of the dose-response curve was performed by GraphPad Prism 8 to calculate the EC_50_ value of the compound.

## Results

### Presence of Putative G-Quadruplex-Forming Sequences in the African Swine Fever Virus Genome

**FIGURE 1:**
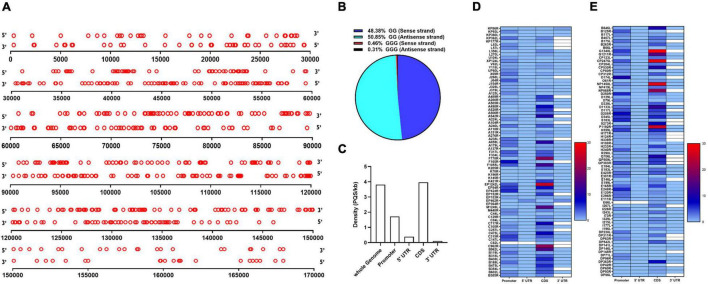
Distribution of putative G-quaruplex-forming sequences (PQSs) in the African Swine Fever Virus (ASFV) genome. **(A)** Schematic representation of the genome-wide distribution of PQSs in the forward (5′ to 3′ orientation) and reverse strands (3′ to 5′ orientation) of the ASFV strain BA71V. Each red circle represents a PQS in its genomic position. The diagram is drawn to scale. **(B)** Quantitative distribution of PQSs with either -GG- or -GGG- islands in the forward and reverse strands of the ASFV genome. **(C)** The density of PQSs in different genomic features as predicted by the QGRS Mapper software. The coding sequences (CDS), 3′ UTRs, and 5′ UTRs were analyzed according to the annotation information as reported by Cackett et al. (2020). The promoter regions were predicted as 1 kb upstream of the transcription start site of each annotated gene. Density represents the number of PQSs per kilobase (kb). **(D,E)** Heat map presentation of the abundance of PQSs in the promoter region, CDS, 3′ UTR, and 5′ UTR regions of individual genes in the ASFV strain BA71V.

The genome of the ASFV BA71V isolate was analyzed using a web-based algorithm, QGRS Mapper, to detect the presence of PQSs. The ASFV BA71V isolate measures 170,101 bp in length encoding 153 genes, and has a guanine/cytosine (GC) content of ∼38.5%. We broadened our search to PQSs comprising of two G-tetrads and a longer loop length of up to 12 nucleotides since it has been demonstrated that two G-tetrads are sufficient to form a G4 and that despite the low stability observed in PQSs with long loop lengths, they are capable of folding into G4s at physiological temperatures (Guédin et al., 2010; Qin et al., 2015). Computational analysis revealed a total of 317 PQSs on the forward strand (Supplementary File 1) and 322 PQSs on the reverse strand (Supplementary File 2), translating to a density of 3.82 PQSs/kb covering 9.52% of the ASFV genome. Similar distribution patterns of PQSs were observed on the forward and reverse strands, with numbers slightly higher on the reverse strand (Figure 1A). 85% of all genes in the ASFV genome had at least one PQS on either strand. The density of G3-PQSs was acutely low, comprising 0.77% of the total predicted PQSs in the entire virus genome, a situation that may be associated with the relatively low viral GC content. G2-PQSs were predominant, accounting for 48.38 and 50.85% on the forward and reverse strands, respectively (Figure 1B).

The number of PQSs in various genomic features, including CDS, 3′ and 5′ UTRs, and promoters, was further analyzed. It was observed that G4s were majorly distributed across the coding sequences and regulatory regions of the ASFV genome with a higher preference for the coding sequence (Figure 1C). Within the coding regions, we identified 593 PQSs in both the forward and reverse strands, at an average density of 3.96 PQSs/kb, making it the region with the highest PQS density in the ASFV genome (Figure 1C). Conservation was assessed in PQSs located in the CDS region of essential genes. The analysis indicated that 89.9% of the PQSs had a conservation score higher than 80% (Supplementary File 3). Some genes had PQSs in their UTRs, including KP86R, EP402R, and D250R (Figures 1D,E). Within the promoter regions, we identified 260 PQSs at an average density of 1.72 PQSs/kb. 68.63% of the gene promoters contained at least one PQS. The frequency of PQSs was significantly elevated in the immediate early gene promoters compared to the late gene promoters. We analyzed the occurrence of PQSs within 500 bp from the TSS. Besides being highly abundant, promoter PQSs exhibited a high conservation rate, with the majority having a conservation score of ≥90% (Supplementary File 4).

### Putative G-Quadruplex-Forming Sequences in Genes Involved in the Entire Lifecycle of African Swine Fever Virus

**FIGURE 2:**
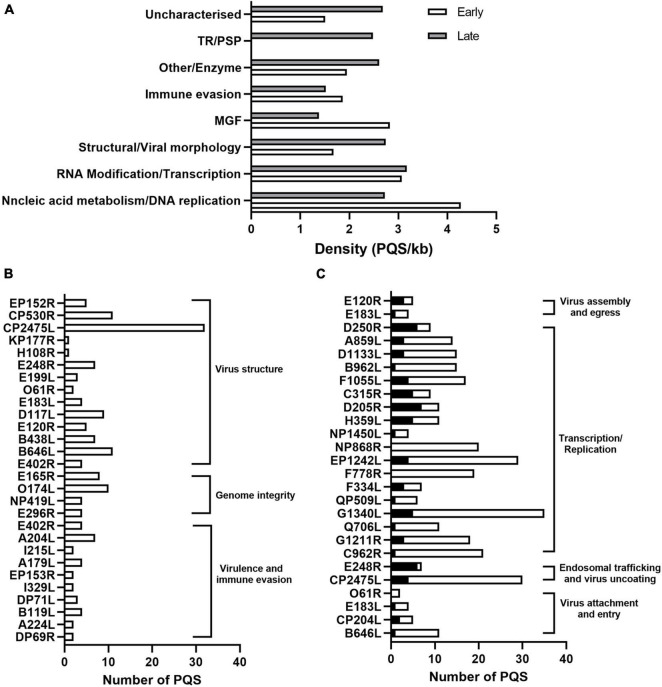
Functional analysis of PQS-containing genes in ASFV. **(A)** Bar graph showing the distribution of PQS-containing genes in their functional categories and their temporal expression phases. The white and gray indicate the early and late expressed genes, respectively. **(B)** Number of PQSs in genes involved in virus structural morphology, genome integrity, virulence, and host immune modulation. **(C)** Distribution of PQSs in genes involved in viral translation and replication. Black columns represent the promoter PQSs, and the white columns represent the CDS PQSs.

We then calculated the number of PQSs in individual genes and presented them according to their functional categories. As shown in Figure 2A, most of the genes involved in translation and replication were enriched with PQSs compared to other functional categories. Early expressed genes involved in DNA replication, transcription, nucleic acid metabolism, and MGF had a significantly high abundance of PQSs compared to late genes in the same functional categories (Figure 2A). On the other hand, in the RNA modification/transcription category, an even distribution of PQSs across early and late genes was noted (Figure 2A).

The distribution of PQSs in the genes involved in viral attachment and internalization was noted. For instance, the pp220 polyprotein encoded by the CP2475L gene, essential for core detachment and release (Alejo et al., 2003), contained 32 PQSs (Figure 2B). Structural proteins B646L, CP204L, and E183L involved in the viral attachment to permissive cells had 11, 5, and 4 PQSs (Figure 2B), respectively.

Multiple genes involved in translation, replication, and DNA repair in the ASFV lifecycle also had multiple PQSs in the promoter and CDS regions. Specifically, G1211R (3 Promoter PQSs and 16 CDS PQSs) that encodes the viral DNA polymerase, P1192R that encodes DNA topoisomerase II (3 Promoter PQSs and 25 CDS PQSs), helicases Q706L (1 Promoter PQS and 10 CDS PQSs) and QP509L (1 Promoter PQS and 5 CDS PQSs), and the subunits of ribonucleotide reductase F334L (3 Promoter PQSs and 4CDS PQSs) and F778R (19 CDS PQSs) all harbored multiple CDS PQSs and/or Promoter PQSs (Figure 2C). Notably, genes involved in transcription and replication were more likely to have promoter PQSs (Figure 2C).

African Swine Fever Virus is transcriptionally independent and, therefore, contains all enzymes and factors required for early mRNA synthesis. Multiple genes associated with this biosynthesis were also found to contain several promoter PQSs and CDS PQSs. These genes included EP1242L (4 Promoter PQSs and 25 CDS PQSs), NP1450L (2 Promoter PQSs and 27 CDS PQSs), H359L (5 Promoter PQSs and 6 CDS PQSs), and D205R (7 Promoter PQSs and 4 CDS PQSs) that comprise the RNA polymerase II complex, C315R (5 Promoter PQSs and 4 CDS PQSs) that encodes eukaryotic transcription factor TFIIB (Rodríguez and Salas, 2013), NUdix hydrolase D250R (6 Promoter PQSs and 3 CDS PQSs) which decaps mRNA, as well as virus-encoded putative RNA helicases, QP509L (1 Promoter PQS and 5 CDS PQSs), F1055L (4 Promoter PQSs and 13 CDS PQSs), B962L (1 Promoter PQS and 14 CDS PQSs), D1133L (3 Promoter PQSs and 12 CDS PQSs), Q706L (1 Promoter PQS and 10 CDS PQSs), and A859L (6 Promoter PQSs and 3 CDS PQSs), which have been speculated to facilitate transcription termination and/or mRNA release (Figure 2C).

As a macrophage-tropic virus, ASFV is prone to oxidative DNA damage due to macrophages producing reactive oxygen species (ROS). Most DNA lesions induced by ROS are repaired by a base excision repair pathway (BER) (Dixon et al., 2019). A dUTPase E165R (3 Promoter PQSs and 5 CDS PQSs), O174L (10 Promoter PQSs) that encodes a reparative DNA polymerase, E296R (3 Promoter PQSs and 1CDS PQS) that encodes an apurinic/apyrimidinic (AP) class II endonuclease, and NP419L (1 Promoter PQS and 3 CDS PQSs) that encodes a DNA ligase, all of which comprise viral enzymes involved in the ASFV BER mechanism contained multiple PQSs (Figure 2B). Targeting these genes with a G4-stabilizing ligand would interfere with the transcription and translation events resulting in an abortive DNA synthesis or production of non-functional proteins.

### Putative G-Quadruplex-Forming Sequences in Genes Associated With African Swine Fever Virus Virulence

Several ASFV genes have been identified as virulence factors that modulate host antiviral responses, including DP69R, A240L, B119L DP71L, DP148R, and a group of 8 MGF360-530 genes (Afonso et al., 2004; Burrage et al., 2004; Neilan et al., 2004). We identified the presence of PQSs in the DP96R, A240L, BII9L, DP71L, and MGF360-530 genes, albeit in low densities, i.e., 2, 7, 4, 2, and 8 PQSs (Figure 2B), respectively. ASFV E183L gene that interacts with microtubules and is required to form viral factories and recruit envelope precursors to virion assembly sites (Dixon et al., 2012; Rodríguez and Salas, 2013) was also found to have 1 Promoter PQS and 3 CDS PQSs (Figure 2C). Targeting PQSs in these genes with stabilizing ligands is likely to impair both the viral mechanisms of pathogenesis and immune evasion strategies.

### Experimental Validation of Folding of Putative G-Quadruplex-Forming Sequences Into Functional G-Quadruplex

**FIGURE 3:**
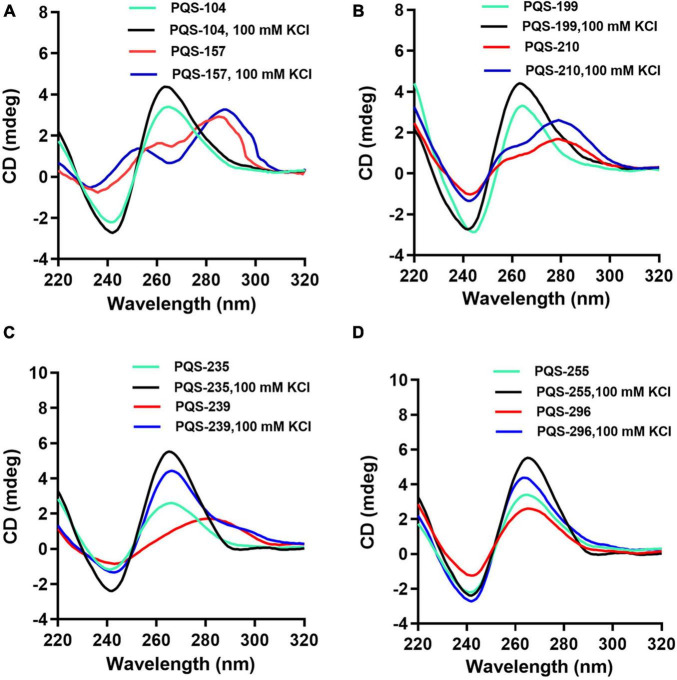
CD spectra analysis of eight conserved PQSs. The oligonucleotides were dissolved in 10 mM Tris–HCl, and CD scanning was performed at a wavelength range of 220–320 nm in the presence and absence of 100 mM KCl. PQS-104 **(A)** and PQS-210 **(B)** folded into a hybrid G4. PQS-157 **(A)**, PQS-199 **(B)**, PQS-235 and PQS-239 **(C)**, PQS-255 and PQS-296 **(D)** formed G4s with parallel conformations.

Since PQSs were predicted in genes involved in the entire lifecycle and virulence of ASFV, 13 highly conserved PQSs (Supplementary Table 4), i.e., PQS-31, PQS-104, PQS-113, PQS-157, PQS-172, PQS-199, PQS-210, PQS-235, PQS-239, PQS-243, PQS-255, PQS-283, and PQS-296, were further assessed for their ability to fold into G4s by CD spectroscopy in the presence and absence of K^+^. Results showed that five of the selected PQSs, i.e., PQS-31, PQS-113, PQS-172, PQS-243, and PQS-283, failed to achieve topological G4 displays (Data not shown). PQS-104 and PQS-210 displayed hybrid/mixed G4 conformation characterized by a negative peak at ∼240 nm and two positive peaks at ∼ 265 and ∼295 nm (Figures 3A,B).

Furthermore, all the other six PQSs, i.e., PQS-157, PQS-199, PQS-235, PQS-239, PQS-255, and PQS-296, adopted a parallel conformation characterized by a negative and a positive peak at ∼240 and ∼265 nm, respectively (Figure 3). In the presence of 100 mM KCl, a marked shift in CD signal was observed in each of these PQSs (Figure 3), indicating visible interactions of K^+^ with these G4s.

### Response of Putative G-Quadruplex-Forming Sequences to G-Quadruplex Ligands

**FIGURE 4:**
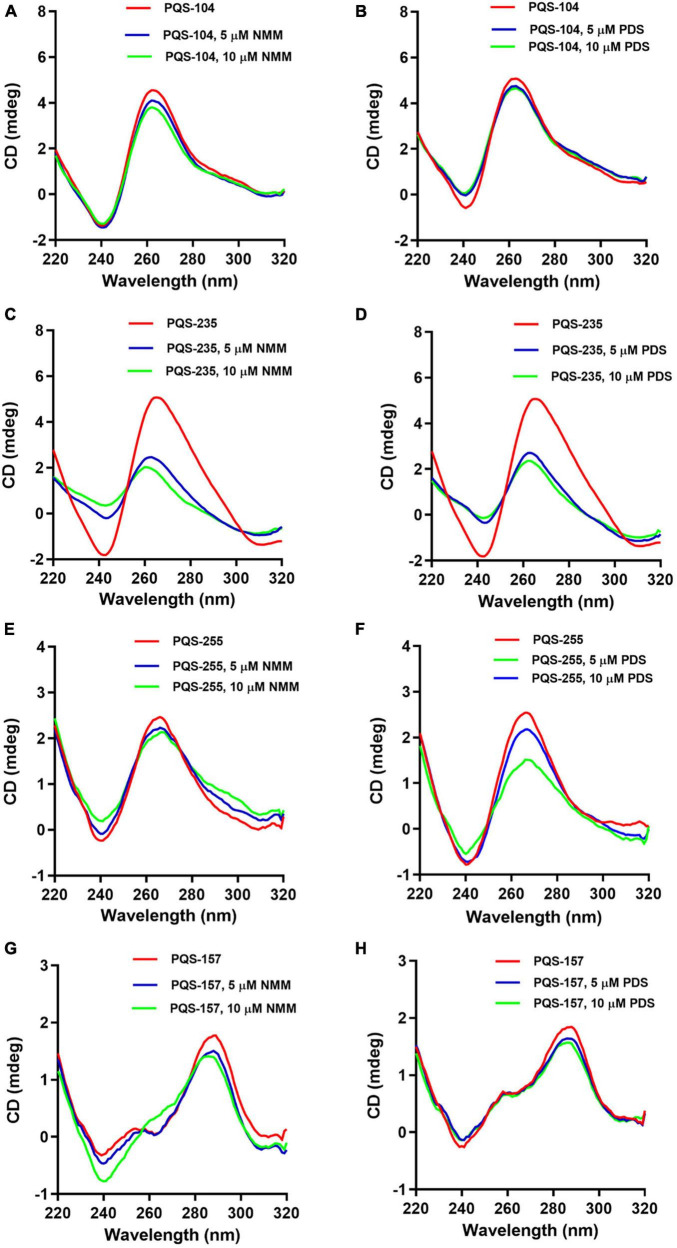
Response of four conserved PQSs to NMM and PDS. CD spectra of PQS-104 **(A,B)**, PQS-235 **(C,D)**, PQS-255 **(E,F)**, and PQS-157 **(G,H)** were recorded in the presence of 5 or 10 μM of each ligand.

Previous studies suggest that G4-binding ligands can stabilize G4 structures by π-stacking onto the G-quartet facets or interaction with the anionic G4 phosphate backbone, loops, and grooves (Largy et al., 2013). To further assess the response of these PQSs to G4-binding ligands, we examined the interaction of all eight PQSs, i.e., PQS-104, PQS-157, PQS-199, PQS-210, PQS-235, PQS-239, PQS-255, and PQS-296, with two typical G4-binding ligands, NMM and PDS. NMM is exceptionally selective for DNA-G4s compared to duplex DNA and shows rare effects on none-G rich DNA, minimizing off-target effects (Adámik et al., 2016). PDS is a highly selective G-quadruplex stabilizing ligand that binds to polymorphic G4 structures regardless of sequence variability (Pagano et al., 2018). To this end, the CD spectra of 5 μM of each PQS was recorded in the presence of 5 or 10 μM of each ligand. It was observed that PQS-199, PQS-210, PQS-239, and PQS-296 were non-responsive to both G4 ligands (Supplementary Figure 1). For PQS-104, PQS-235, and PQS-255, the addition of NMM resulted in an upward shift of the negative peak (∼240 nm) and a downward shift in the positive peak (∼265 nm) which corresponded with an increase in G4 ligand concentration (Figures 4A–F). As shown in Figures 4G,H, PQS-157 displayed a downward shift in the two positive peaks (∼265 and ∼295 nm) and a minimal upward shift in the negative peak (∼240 nm). As excepted, similar interaction patterns were observed between these PQSs and PDS (Figure 4). These results are indicative of a favorable interaction of these PQSs with the G4-stabilizing ligands.

**FIGURE 5:**
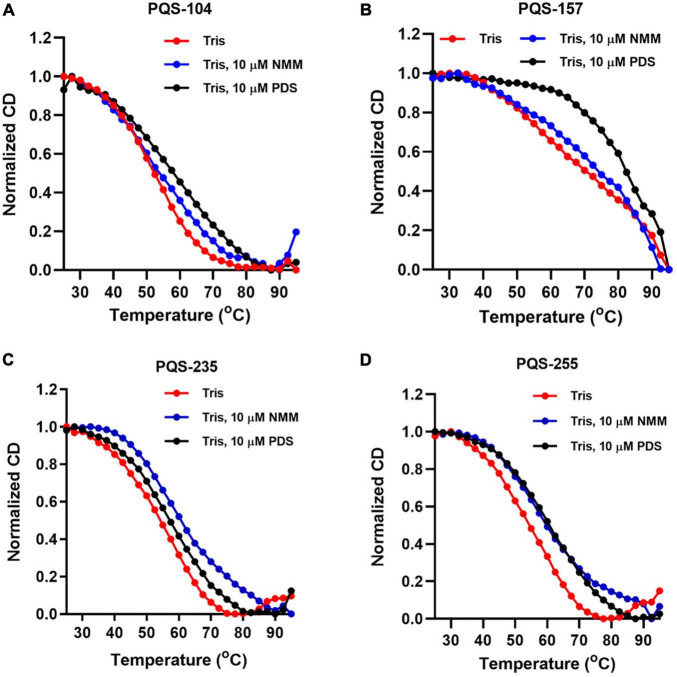
CD melting curves of four conserved PQSs. The CD melting curves of PQS-104 **(A)**, PQS-157 **(B)**, PQS-235 **(C)**, and PQS-255 **(D)** were recorded in the presence or absence of 10 μM NMM and PDS.

Next, the stabilization of these G4 structures, i.e., PQS-104, PQS-235, PQS-255, and PQS-157, by NMM and PDS was further characterized by CD melting spectra analysis as described previously (Kelly and Price, 2000). In this regard, the CD melting spectra of each PQS-ligand mixture was recorded at 265 nm over a temperature range of 25–95°C with an increasing rate of 5°C/min. As shown in Figure 5, all four PQSs were responsive to stabilization by NMM and PDS, as indicated by a ligand-induced increase in melting temperature (ΔT_m_). Specifically, an increase of 4.1–9.7°C and 5.7–13.9°C was observed in these four PQSs in the presence of NMM and PDS (Table 1), respectively.

**TABLE 1:** G4 ligand-induced changes in melting temperature for PQSs in the presence of 10 μM NMM or PDS.

PQS	Tm in Tris (°C)	With NMM		With PDS	
		Tm (°C)	ΔTm (°C)	Tm (°C)	ΔTm (°C)
PQS-104	44.9	49.0	4.1	50.6	5.7
PQS-157	68.2	72.4	4.2	82.1	13.9
PQS-235	48.7	58.4	9.7	55.2	6.5
PQS-255	49.3	56.2	6.9	56.6	7.3

### Effect of G4-Stabilizing Ligands on Gene Expression in 293T Cells

**FIGURE 6:**
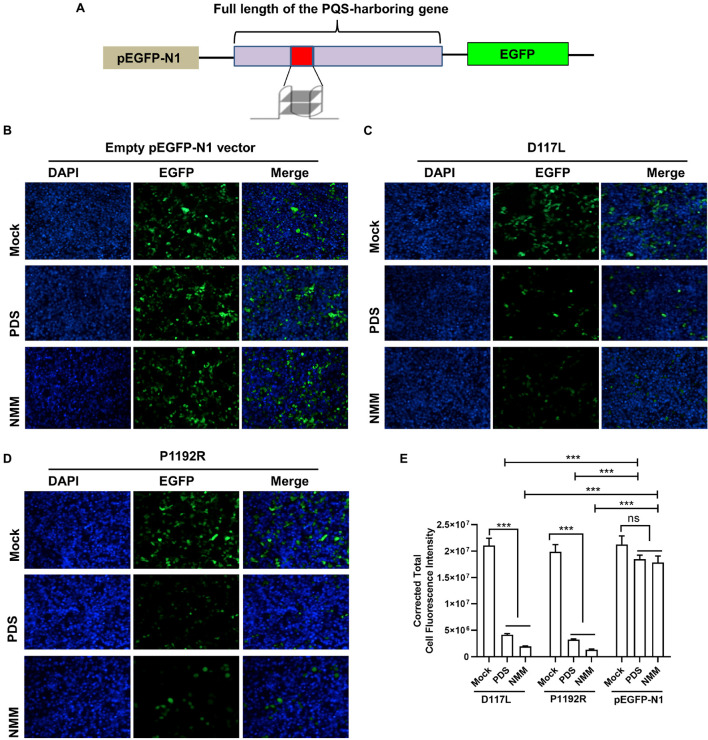
The effect of G4 stabilizing ligands on gene expression. **(A)** Schematic illustration of PQS-recombinant plasmids construction. Plasmids containing empty pEGFP-N1 vector **(B)**, D117L **(C)**, or P1192R **(D)** were transfected into 293T cells in the presence or absence of 20 μM of each ligand. After 24 h of incubation, the expression of EGFP in each well was assessed by confocal microscopy. **(E)** Quantitative fluorescence signal using corrected total cell fluorescence method for EGFP in cells transfected with plasmids containing D117L, P1192R, and the empty vector. ns, not significant; ***p < 0.001.

To further confirm whether PQS-containing genes could respond positively to G4-stabilizing ligands, an EGFP reporter system was employed to investigate the expression of PQS-containing genes in the presence of G4-stabilizing ligands. Since PQS-235 and PQS-255 showed good response to NMM, their original genes, i.e., D117L and P1192R, were inserted upstream of the EGFP-coding sequence in a pEGFP-N1 vector and transfected to 293T cells (Figure 6A). Treatment with NMM and PDS showed rare influence on the expression of EGFP in 293T cells (Figure 6B). As expected, the expression of EGFP was significantly inhibited in cells transfected with either D117L (Figure 6C) or P1192R (Figure 6D) in the presence of NMM and PDS compared to the vector-transfected control cells, which was further confirmed by fluorescence intensity calculation analysis (Figure 6E). These results showed that both PQSs could be stabilized by NMM and PDS in cells and lead to the downregulation of the downstream EGFP gene.

### N-Methyl Mesoporphyrin Inhibits the Replication of African Swine Fever Virus in PAM Cells

**FIGURE 7:**
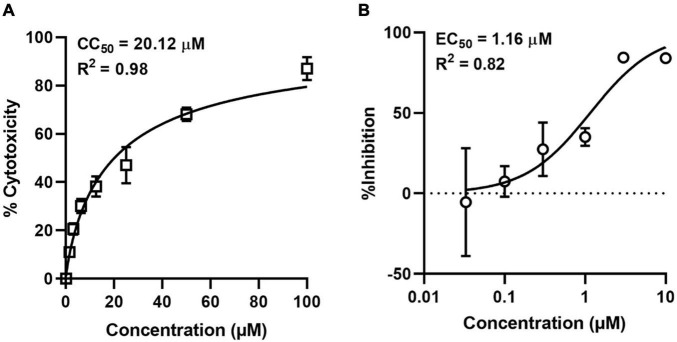
Cytotoxicity and antiviral activity of NMM against ASFV in cell culture. **(A)** Cytotoxicity of NMM in PAM cells was determined by MTT Assay. **(B)** Antiviral activity of NMM against ASFV. PAM cells were infected with ASFV strain Wuhan 2019-2 at an MOI of 0.1 in the presence of various doses of NMM (0, 0.03, 0.1, 0.3, 1, 3, and 10 μM). 72 h post-infection, viral yield in the culture supernatant was quantified by qRT-PCR.

Since the existence of hundreds of PQSs in the whole genome of ASFV had been predicted and G4 ligands could block the expression of PQS-containing genes, we asked whether these PQSs could serve as potential antiviral targets by G4 ligands. To address this, we tested the antiviral activity of NMM against ASFV in PAM cells. First, we determined the cytotoxicity of NMM in PAM cells by MTT assay. The compound showed dose-dependent toxicity against PAM cells with a 50% cytotoxic concentration of 20.12 μM (Figure 7A). For the antiviral assay, PAM cells were infected with ASFV at an MOI of 0.1 in the presence of various concentrations of NMM (0–10 μM). After 72 h of infection, the viral DNA in the supernatant was quantified by qPCR with primers targeting the P72 gene. Our results showed that NMM inhibits the replication of ASFV in a dose-dependent manner, with a 50% effective concentration (EC_50_) value of 1.16 μM (Figure 7B), suggesting that G4 ligands could serve as potential antiviral agents against ASFV.

## Discussion

In viruses, the occurrence of G4s has been reported in the Nipah virus (Majee et al., 2020), Human Immunodeficiency Virus (Ruggiero et al., 2019), Human Herpes Simplex Virus (Artusi et al., 2015), Hepatitis C virus (Jaubert et al., 2018), SARS-CoV-2 (Zhang R. et al., 2020), and their targeting by small-molecule ligands with consequent antiviral effects. A recent analysis of putative PQSs in seven viral taxa infecting distant eukaryotic hosts demonstrated co-evolution of the viruses and host G4s. Besides, PQSs were highly conserved despite the high recombination rates in viruses, highlighting the fundamental roles of G4s in the viral life cycle (Puig Lombardi et al., 2019). In the present study, we analyzed the distribution of PQSs in the ASFV genome and investigated the possibility of a G4-targeted antiviral approach *in vitro*.

Using computational analysis, we identified 317 PQSs on the forward strand and 322 PQSs on the reverse strand of the ASFV genome. The number of PQSs with ≥3 G-runs (-GGG-) found on either strand was surprisingly low. G2 type of PQS was predominant in both strands. Multiple studies have proven that PQSs consisting of four contiguous GG runs can fold into stable two-quartet G4s (Qin et al., 2015). Biophysical and functional characterization studies of two-quartet G4s have demonstrated critical roles associated with these G4 motifs. For example, in the polyamine biosynthesis pathway, G2 motifs could detect and regulate cellular polyamine levels, forming feedback loops (Lightfoot et al., 2018). In a separate study, G_2_-PQS located in the 3′ UTR of the *IE180* gene in pseudorabies virus could regulate the expression of the gene, thus regulating viral replication. This motif serves as a post-transcription regulation element in the presence of G4-binding ligands (Zhang Y. et al., 2020). Conservation analysis discovered some extremely conserved PQSs in both the coding and promoter regions of the ASFV genome. Conserved PQSs highlight their crucial roles in virus survival and may serve as universal targets for ASFV antiviral drugs.

Among the identified PQSs, we selected 13 of them located in the CDS regions for characterization studies and to assess the effects of G4 stabilizers on them. Biochemical characterization demonstrated that 8 PQSs could form stable G4s in the presence of K^+^, and 4 of them could be stabilized by G4 ligands. Of the 4, 2 G4s in the coding regions of P1192R and D117L could modulate gene expression in the presence of G4 stabilizers. Besides the characterized sequences, D117L and P1192R had 4 and 24 PQSs in their coding sequences, respectively. PQSs in these genes could have possibly served as multiple targets, thereby contributing to the excellent repression of gene expression in the presence of G4 ligands *in vitro*. Our experimental screening results show that not all PQSs can fold into functional G4s and that not all G4s are of clinical relevance in the context of antiviral drug development.

Of particular interest is the presence of a functional G4 in the P1192R gene, given its essential roles in the viral replication, transcription, and genome processes. In a study involving Top2p, a *Saccharomyces cerevisiae* DNA topoisomerase, it was noted that cells could complete the replication process in the absence of the gene. However, the cells die just after entering the mitotic phase. In the presence of an inactive form of Top2p, cells fail to complete DNA replication at the convergence site of the replication fork (Baxter and Diffley, 2008). Given that P1192R is a key component of the ASFV replication machinery, a replication-defective vaccine has been proposed by generating mutations/deletions within this gene (Coelho and Leitão, 2020). Based on this model, targeting the G4s in the ASFV DNA topoisomerase gene with G4-stabilizing ligands is a promising approach in developing an antiviral drug for ASFV.

Breakthroughs have been realized in the treatment of human infections by targeting clinically relevant genes in pathogens. TMPyP4, an alkyl derivative, could specifically identify and bind to G4s in the 5′ UTR of *KRAs* mRNA, thereby regulating its expression in pancreatic cancer cells (Cogoi and Xodo, 2006). Stabilization of the 5′ UTR of the *NRAS* proto-oncogene by the pyridine-2, 6-bis-quinolinodicarboxamide derivative suppresses its translation (Kumari et al., 2007). This gene serves as an on and off switch that regulates cell differentiation and apoptosis. In another study, G4 ligands have been shown to forestall the incorporation of the HIV genome into the host genome by binding to and stabilizing G4s in the multi-domain HIV integrase enzyme (Cree and Kennedy, 2014; Bala et al., 2018).

Altogether, there is so much proof that G4 ligands and their derivatives can bind to and block clinically relevant proteins, thereby preventing the progression of human viruses and suppressing cancer-aggravating genes. Based on our current study, we extend this validation to animal viruses such as ASFV. Besides direct targeting of viral PQSs, G4-binding ligands have been known to induce a DNA damage response (DDR), suggesting a dual mechanism of action (De Magis et al., 2019). They can target the virus and activate the hosts’ cellular stress response pathways. Quarfloxin, a potent G4 ligand that targets rDNA G4 complexes, got to clinical phase II trials for cancer treatment (Asamitsu et al., 2019).

The inclusion of G4 research in the agricultural sector holds a promising approach in managing devastating diseases such as ASFV. Although the *in vivo* efficacy of G4 ligands against ASFV still needs to be established, the current data clearly showed that several PQSs in the ASFV genome could fold into functional and stable G4s, which G4 ligands, such as NMM could stabilize. Therefore, G4 ligands may represent potential candidates for anti-ASFV drugs.

## Data Availability Statement

The original contributions presented in the study are included in the article/Supplementary Material, further inquiries can be directed to the corresponding author/s.

## Author Contributions

HY: conceptualization, writing—review and editing, and funding acquisition. HY, EM, FM, and HL: methodology. EM, FM, and HL: investigation. HY and EM: formal analysis. EM, MJ, FM, and HL: data curation. EM: writing—original draft. HY and HW: supervision. HW: resource. All authors contributed to the article and approved the submitted version.

## Conflict of Interest

The authors declare that the research was conducted in the absence of any commercial or financial relationships that could be construed as a potential conflict of interest.

## Publisher’s Note

All claims expressed in this article are solely those of the authors and do not necessarily represent those of their affiliated organizations, or those of the publisher, the editors and the reviewers. Any product that may be evaluated in this article, or claim that may be made by its manufacturer, is not guaranteed or endorsed by the publisher.

## Footnotes

1
https://bioinformatics.ramapo.edu/QGRS

2
https://www.ebi.ac.uk/tools/msa/mafft/
