# Supplementary material for: Comprehensive Analysis of G-Quadruplexes in African Swine Fever Virus Genome Reveals Potential Antiviral Targets by G-Quadruplex Stabilizers
Source: Front Microbiol. 2021 Dec 16;12:798431. doi: 10.3389/fmicb.2021.798431 (PMC8718096; doi:10.3389/fmicb.2021.798431)
Supplement: Supplementary file 1 [file Data_Sheet_1.pdf]

Supplementary Material

Comprehensive analysis of G-quadruplexes in African Swine Fever Virus genome reveals potential antiviral targets by G-quadruplex stabilizers

Elishiba Muturi[1,2], Fei Meng[1], Huan Liu[1,2], Mengwei Jiang[1], Hongping Wei[1,2*], Hang Yang[1,2*]

[1]CAS Key Laboratory of Special Pathogens and Biosafety, Centre for Biosafety Mega-Science, Wuhan Institute of Virology, Chinese Academy of Sciences, Wuhan 430071, China.

[2]University of Chinese Academy of Sciences, Beijing 100049, China

Table S1. Accession numbers of ASFV isolates used in this study

Genotype	Isolate
Genotype I	ASFV_OURT_88_3_AM712240.1
	ASFV_BA71_KP055815
	ASFV_BA71V_NC001659
	ASFV_NHV_KM262844
	ASFV_L60_KM262844
	ASFV_E75_FN557520
	ASFV_Benin_97_1_AM712239
	ASFV_47_Ss_2008_KX354450
	ASFV_26544_OG10_KM102979
Genotype II	ASFV_China_2018_AnhuiXCGQ_MK128995
	ASFV_Pol17_05838_C220_MG939589
	ASFV_Pol17_04461_C210_MG939588
	ASFV_Pol17_03029_C210_MG939587
	ASFV_Pol16_29413_o23_MG939586
	ASFV_Pol16_20186_o9_MG939584.1
	ASFV_Pol16_20186_o7_MG939583
	ASFV_Belgium_2018_1_LR536725.1
	ASFV_Maldova_2017_1_PRJEB33279
	ASFV_Georgia_2008_2_MH910496
	ASFV_Georgia_2007_1_FR682468.2

	ASFV_Pol_2015_Podlaskie_MH681419<br>ASFV_Georgia_2008_2_MH910495<br>ASFV_Kashino_04_13_KJ747406<br>ASFV_Estonia2014_LS478113<br>ASFV_Odintovo02_14_KP843857<br>ASFV_SY18_MH766894<br>ASFV_Georgia_2007_1_FR682468.1
Genotype III	ASFV_Warmbaths_AY261365.1
Genotype IV	ASFV_Warthog_AY261366
Genotype V	ASFV_Tengani61_AY2613364.1
Genotype VIII	ASFV_Malawi_AY261361.1
Genotype IX	ASFV_N10_MH025916<br>ASFV_R25_MH025918<br>ASFV_Ken06bus_KM111295<br>ASFV_R8_MH0225919<br>ASFV_R35_MH025920<br>ASFV_R7_MH025917
Genotype X	ASFV_Ken05_Tk1_kM111294<br>ASFV_Kenya_1950_AY261360
Genotype XII	ASFV_Mkuzi_1979_AY261362.1
Genotype XX	ASFV_PR4_AY2613631

Table S2. Sequence of PQS oligonucleotides used in this study with the strand position and the G-Scores as predicted by QGRS Mapper.

PQS	Position	Strand	Length (bp)	Sequence	G Score	Conservation (%)	Gene	Gene Function
PQS-31	34164	+	14	ggggaggagccagg	31	98.2	A859L	Helicase superfamily I
PQS-104	74087	+	23	ggcttaaggggcggggatatggg	36	98.4	C962R	DNA primase
PQS-113	77328	+	11	ggtggcggagg	36	100.0	B962L	Helicase superfamily II
PQS-157	93995	+	26	ggtagaaaggcaatccggttcaccgg	36	100.0	G1340L	VV A8L-like transcription factor
PQS-172	97021	+	38	gggaaccgttccggacccaaatcaggcaaacgacgtgg	36	98.9	G1211R	DNA polymerase family B
PQS-199	112021	+	27	ggcacttgatggttcaagtggtggagg	32	99.7	O61R	Attachment protein
PQS-210	115767	+	20	ggttgcggtggtggaaacgg	36	99.9	NP1450L	RNA polymerase subunit 1
PQS-235	127251	+	23	gggaaatggtggtgaggctcagg	34	98.7	D117L	Required for progression of precursor membranes to icosahedral intermediates
PQS-239	128082	+	26	ggtggtctggctgggaggaagaccgg	36	99.9	D205R	RNA polymerase subunit 5
PQS-243	130246	+	23	ggatatggtaaaggtatataagg	33	99.9	S273R	SUMO-1-like protease

PQS-255	133084	+	25	ggcacctgggaggaaaggatgcagg	35	98.0	P1192R	DNA topoisomerase type II
PQS-283	145693	+	23	ggtttagaggtacctggttgtgg	34	99.5	E183L	Binds to LC8 of dynein light chain; Involved in virus entry
PQS-296	150524	+	18	ggtggtgaaggggaatgg	32	99.9	E248R	Possible component of redox pathway; Required for disulphide bond formation

Table S3. Primers used for cloning of PQS into a pEGFP-N1 plasmid.

PQS	Gene	Forward primer (5'-3')	Reverse primer (5'-3')
PQS-104	C962R	tatagctagcatgcgcgaggaatcatgggaagac	tatagtcgactgccattcccaccatttatttttg
PQS-157	G1340L	tatagctagcatggatttccaaaatgac	tatagtcgactgaaacttatttttacactaataatttc
PQS-235	D117L	atatgctagcatggacactgaaacgtc	tatagtcgactgtgaatgcgcaagttc
PQS-255	P1192R	atataagcttatggaagcgtttgaaatc	tataggtaccgtatgaaatttccactgag

Table S4. Sequence logos constructed by WebLogo software for 13 PQSs.

PQS	Sequence
PQS-31	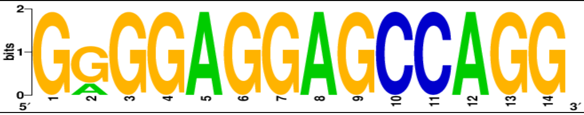
PQS-104	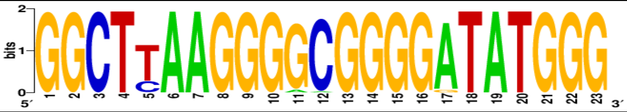
PQS-113	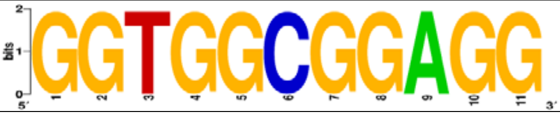
PQS-157	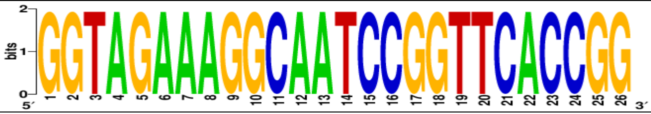
PQS-172	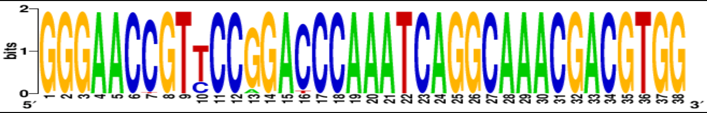
PQS-199	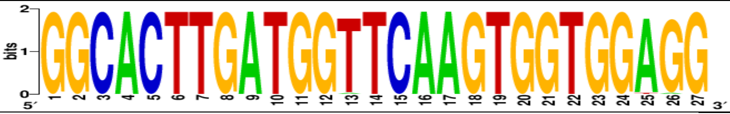
PQS-210	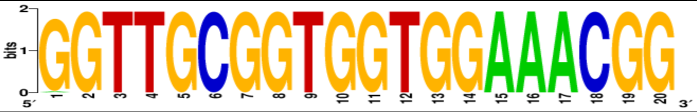
PQS-235	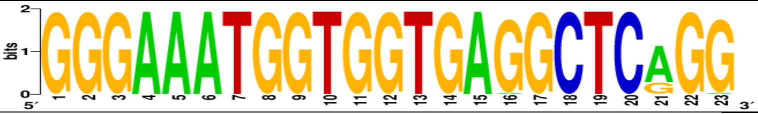
PQS-239	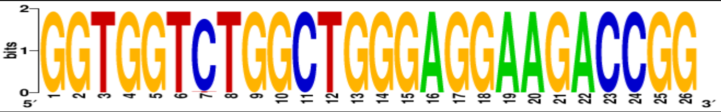
PQS-243	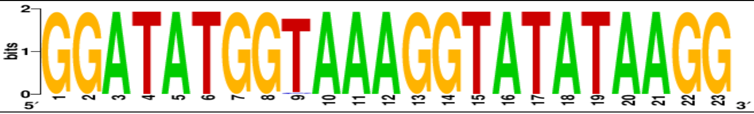
PQS-255	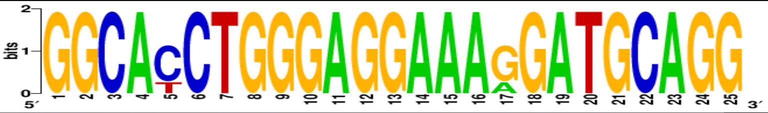
PQS-283	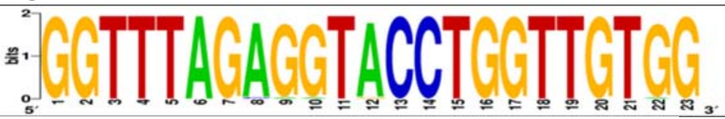
PQS-296	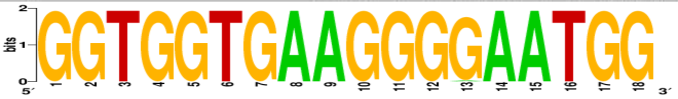

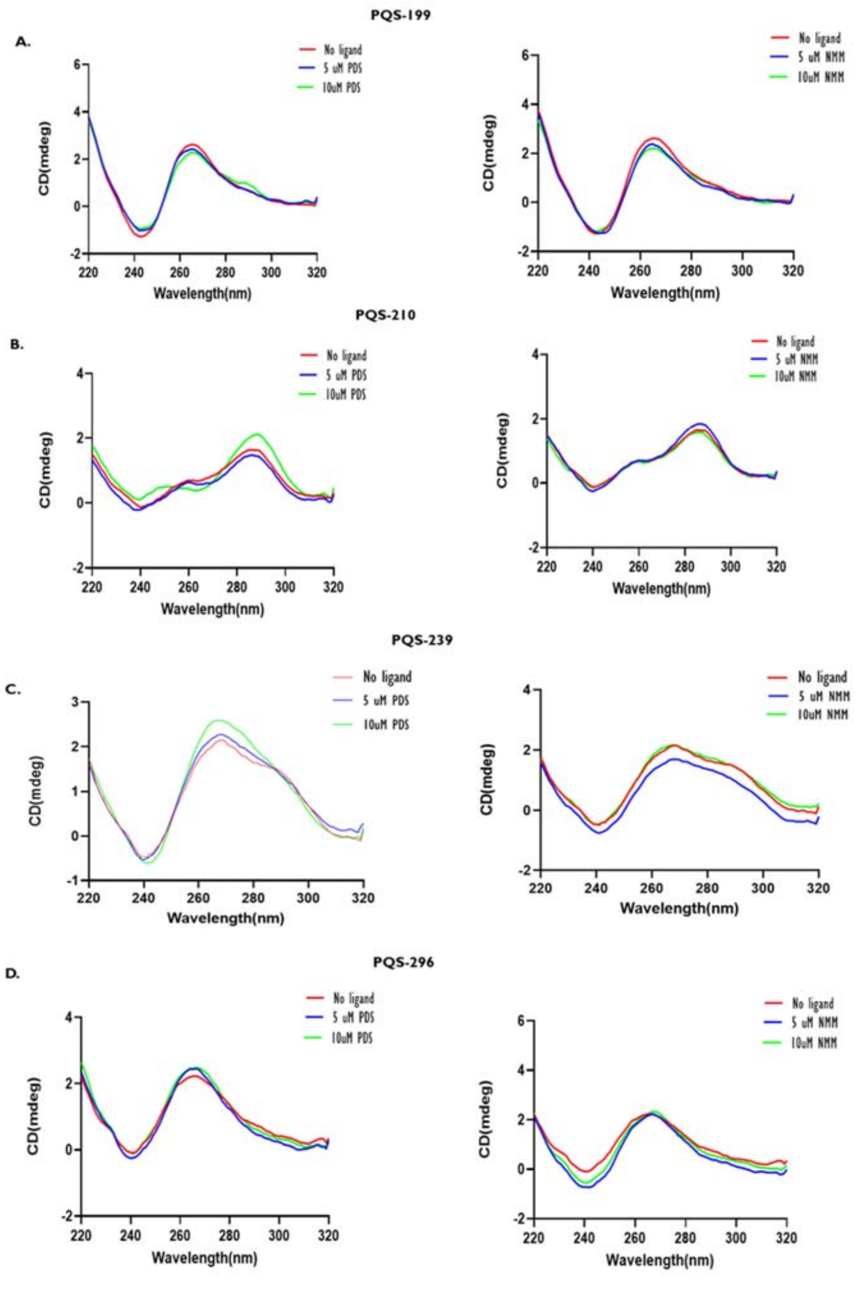

Figure S1. Responses of PQS-199, PQS-210, PQS-239, and PQS-296 to G4 ligands. CD spectra of PQS-199 **(A)**, PQS-210 **(B)**, PQS-239 **(C)**, and PQS-296 **(D)** in the presence or absence of 5 µM or 10 µM NMM and PDS.
